# Isolation, characterization and virulence assessment of Type 1d CP bovine viral diarrhea virus originating from Inner Mongolia, China

**DOI:** 10.3389/fvets.2025.1554986

**Published:** 2025-02-27

**Authors:** Luyao Hao, Fengmiao Zhao, Ting Guo, Yuan Gao, Yongqing Hao

**Affiliations:** ^1^College of Veterinary Medicine, Inner Mongolia Agricultural University, Hohhot, China; ^2^Key Laboratory of Clinical Diagnosis and Treatment of Animal Diseases, Ministry of Agriculture, National Animal Medicine Experimental Teaching Center, Hohhot, China; ^3^Inner Mongolia Academy of Agricultural and Animal Husbandry Sciences, Hohhot, China

**Keywords:** isolation, Type 1d CP bovine viral diarrhea virus, pathogenicity, prevention, model laboratory animals

## Abstract

In recent years, bovine viral diarrhea virus (BVDV) has been associated with increased respiratory and gastrointestinal diseases in cattle. Comprehensive monitoring and investigations into the virus's pathological features are crucial for developing effective prevention strategies. This study investigated BVDV prevalence and pathogenicity in farms undergoing elimination protocols, with a focus on characterizing a novel Cytopathic Bovine Viral Diarrhea Virus (CP-type BVDV) strain (HH839) isolated from a symptomatic calf in Hohhot, Inner Mongolia. During 2021 and 2022, 103 bovine samples were screened for BVDV via nucleic acid detection. Positive cases underwent viral isolation using MDBK cells. The HH839 strain was analyzed for cytopathic effects, ultrastructure (electron microscopy), antigenicity (serum neutralization), and genetic lineage (whole genome sequencing). Pathogenicity of Cytopathic Bovine Viral Diarrhea Virus (CP-type BVDV) infected group, Noncytopathic Bovine Viral Diarrhea Virus (NCP-type BVDV) infected group, and the mixed-infection group of CP-type and NCP-type BVDV was evaluated in New Zealand White rabbits, with viral distribution and histopathological damage assessed in multiple organs. We identified 33 positive BVDV nucleic acid cases, resulting in a positivity rate of 32.04%. Five strains of NCP-type BVDV were isolated, yielding a 15.15% separation rate, alongside one strain of CP-type BVDV with a separation rate of 3.03%. The CP strain HH839 was isolated from a severely symptomatic calf in Hohhot, Inner Mongolia. The HH839 strain demonstrated significant cytopathic effects in MDBK cells, including cellular crumpling and syncytia formation, with a concentration of 5.23 log_10_TCID_50_/0.1 mL. Electron microscopy revealed a spherical morphology with a diameter of 40–60 nm. Genetic analysis indicated a close relationship with the BVDV FBS-D8 strain from the BVDV-1d subtype. Pathogenicity trials showed slight fever and minor body weight loss in infected subjects, with BVDV detected in the trachea, lungs, spleen, and small intestines, predominantly in the spleen. The isolation of HH839, a pathogenic CP-type BVDV-1d strain, underscores the coexistence of multiple BVDV biotypes in regional cattle populations. Enhanced pathogenicity observed in mixed infections highlights complex viral interactions. These findings emphasize the necessity for sustained surveillance and biotype-specific control strategies to mitigate BVDV-associated economic losses in livestock industries.

## 1 Introduction

Bovine viral diarrhea virus (BVDV) is a member of the pestivirus genus and the Flaviviridae family. The pestivirus genus currently comprises four species: *Pestivirus bovis* (commonly known as BVDV-1), *Pestivirus tauri* (BVDV-2), *Pestivirus ovis* (Border disease virus, BDV), and *Pestivirus suis* (Classical swine fever virus, CSFV) (1, 2). BVDV is one of the major causes of bovine respiratory disease complex (BRDC). It is widespread globally and poses a serious threat to the livestock industry (3). The epidemiological characteristics of BVDV provide important scientific basis for reducing BRDC—related losses worldwide, promoting vaccine development, and improving diagnostic techniques (4). Similarly, in China, the spread of BVDV has exacerbated economic losses and affected the productivity of beef cattle and dairy cows (5, 6). It was first isolated in China in 1980 (7), followed by its spread in China. In total, the pooled prevalence of BVDV in yaks in China was 36.0% from 1987 to 2019 (8). The pooled BVDV prevalence in dairy cattle in China was estimated to be 53.0% (9). The rate of BVDV in Inner Mongolia was 44.70% from 2018 to 2019 (10).

BVDV is classified into two main species: BVDV-1 and BVDV-2, as well as a third group of related viruses, known as HoBi-like pestivirus, sometimes referred to as BVDV-3 (11, 12). The 5′UTR, Npro, and E2 regions are mainly used for subtypes identification (13). To date, a total of 22 BVDV-1 subtypes (1a−1v), a suspected new subtype 1w, and four BVDV-2 subtypes (2a−2d) have been reported worldwide (13, 14), which predominate in different countries. In China, subtypes identified in cattle include 1a, 1b, 1c, 1d, 1m, 1o, 1p, 1q, 1u, 1v, 2a, and 2b (15). In recent years, the main dominant subtypes circulating in China have been 1a, 1c, and 1m. There are differences in immune responses among these genetic subtypes, which may affect the effectiveness of vaccines and diagnostic reagents. Therefore, the isolation and identification of BVDV, along with genetic evolution analysis, may contribute to the development of local preventive strategies and diagnostic tools, helping in the prevention and control of BVDV (16). The BVDV genome is 12.3–12.5-kb-long consisting of ssRNA, and the open reading frame includes 11–12 proteins: Npro, C, Erns, E1, E2, P7, NS2–3 (or NS2, NS3), NS4A, NS4B, NS5A, and NS5B from 5′ to 3′ (17). BVDV can be classified into cytopathic (CP) and noncytopathic (NCP) biotypes, based on their ability to induce visible cytopathic effects in cultured cells (18). However, the relationship between these biotypes is more complex, as the presence of cytopathic effects can depend on factors such as viral strain, host cell type, and environmental conditions. BVDV infection causes immune dysfunction (19). Different subgenotypes and biotypes of BVDV lead to different clinical symptoms (20, 21).

In this study, a calf in a cattle farm in Hohhot suffered from severe fever, cough, and diarrhea, suspected of bovine viral diarrhea. We report the isolation and identification of a HH839 strain of BVDV from a herd of calf that had severe disease. We used the New Zealand white rabbit infection model to perform an animal regression experiment and assess pathogenicity. This study is important to drive vaccine development and determine the presence of BVDV in Inner Mongolia.

## 2 Materials and methods

### 2.1 Clinical cases and sampling

Sixty ear tissue samples and 43 anal swabs were collected from calves at cattle farms with BVDV eradication plans in Inner Mongolia, China, from June 2021 to January 2022. Ear tissue samples were randomly collected by local veterinarians from cattle that were suspected to be persistently infected (PI) with BVDV, based on factors such as herd history, previous exposure risks, or other clinical surveillance indicators, despite the fact that these animals did not show clinical symptoms. Anal swabs were collected from cattle exhibiting varying degrees of clinical symptoms, such as fever, rhinorrhea, cough, and diarrhea. All the samples obtained for this experiment were collected with permission from the farm owner and all samples were immediately transported to the laboratory at low temperature after collection. The samples with positive BVDV detected by RT-PCR were stored at −80°C.

### 2.2 Animals

Twenty female New Zealand white rabbits, weighing between 1.5 to 2.2 kg and aged between 2 to 3 months, were purchased from Beijing Xinogu Valley Biotechnology Co., Ltd. During the experimental process, all animals had access to *ad libitum* food and water, and were housed in the clean animal facility of Inner Mongolia Agricultural University, with good ventilation and a constant room temperature (24°C ± 0.5°C).

### 2.3 Detection of BVDV in ear tissues samples

The collected tissues were ground, and anal swabs were thawed and frozen three times. Total RNA was extracted using the AxyPrep™ Multisource Total RNA Miniprep Kit (Axygen, Corning, China) according to the manufacturer's instructions and stored at −80°C until testing. For cDNA synthesis, 1 μg of total RNA was used as the template, and reverse transcription was carried out using the HiScript III Reverse Transcriptase (Vazyme, China) according to the manufacturer's protocol. The reverse transcription reaction was conducted in a final volume of 20 μl, with the following components: 4 μl of 5× HiScript III RT SuperMix, 1 μl of oligo (dT) 20 primer, 1 μl of random hexamers, and the appropriate amount of RNA template, adjusted to 20 μl with nuclease-free water. After reverse transcription, the cDNA was amplified by PCR using a set of primers specific for the 5′RTR region: 190F (5′-GRA-GTC-GTC-ART-GGT-TCG-AC-3′) and V326 (5′-TCA-ACT-CCA-TGT-GCC-ATG-TAC-3′) (22). The PCR reaction mixture (25 μl final volume) included 3 μl of cDNA template, 12.5 μl of 2× Taq Master Mix, 1 μl of forward primer (10 μM), 1 μl of reverse primer (10 μM), and 7.5 μl of nuclease-free water. The reaction was carried out using one unit of Taq polymerase per reaction.

### 2.4 Isolation and identification of BVDV

These samples were diluted in Dulbecco's modified Eagle medium (DMEM) maintenance fluid containing 1% penicillin-streptomycin and clarified by low-speed centrifugation (4,000*g* for 10 min). Subsequently, the supernatants were filtered through 0.22-μm pore membrane filters (Merck Millipore Ltd., Burlington, MA, USA). One milliliter of the supernatant was transferred to Madin-Darby bovine kidney (MDBK) cells and incubated at 37°C in 5% CO_2_ for 2 h. Thereafter, a cell maintenance fluid containing 2% fetal bovine serum (FBS) was added to bring the total volume to 5 ml. The cultures were then incubated at 37°C in a 5% CO_2_ incubator. Cytopathic effects (CPE) were observed daily, and the cultures were collected after 5 days. Non-cytopathic viruses, which do not induce visible CPE, were identified by molecular methods, RT-PCR, to detect viral RNA in the culture supernatant.

### 2.5 Electron microscopy observation of BVDV

After freezing and thawing the virus cultures thrice, cells were blown into the centrifuge tube using a straw, inhaled into the centrifuge tube, and centrifuged (no more than 4,000*g*) for ~2 min; finally, the supernatant was discarded. Finally, glutaraldehyde fixative was added (2.5%) at room temperature; the cell mass was gently picked up a, suspended in the fixative, and fixed at room temperature in the dark for 30 min. BVDV particles from infected MDBK cells were visualized using transmission electron microscopy (HITACHI HT7650TEM).

### 2.6 Direct immunofluorescence

IFAs tested the expression of viral proteins in BVDV-infected MDBK cells, which were inoculated with HH839 at a multiplicity of infection (MOI) of 0.05. Post-incubation, the cells were fixed with 4% paraformaldehyde. Each well received 150 μl of BVDV FITC antibodyPolyclonal (VMRD; No. CJ-F-BVD-10 μl) and was incubated for 1 h at 37°C in the dark. After washing with PBS, 150 μl of 1× DAPI staining solution was applied for 15 min. Excess liquid was removed, and a 50% glycerol-containing DAPI (Roche) solution (40 μl/well) was added for another 15-min incubation. Following two PBS washes, the plate was observed under an inverted fluorescence microscope, and images were captured (Zeiss, Oberkochen, Germany) (23).

### 2.7 Serum neutralization

In the assay, the isolated virus was mixed with the BVDV-positive serum and incubated at 37°C for 1 h to allow for the formation of immune complexes. The serum-virus mixture was then added to a monolayer of susceptible cells in a 96-well plate and incubated for 7 days, depending on the cytopathic effect (CPE) observed.

The presence of cytopathic effects was monitored daily (24).

### 2.8 TCID_50_ titration of viral isolates

Titration of TCID_50_ for HH839 isolates was performed using 96-well plates. The viruses were diluted at 10× serial dilutions with serum-free DMEM from 10^−1^ to 10^−8^ and used to infect the wells for each dilution with 100 μl, next, these were placed in a 37°C 5% CO_2_ incubator for 1 h, after which 100 μl of cell maintenance fluid was added and placed into the incubator for further incubation. Subsequently, the cytopathic effects on the MDBK cells were observed and counted once at 24 h intervals, and half of the tissue cell infection volume (TCID_50_) was calculated using the Reed-Muench method (25).

### 2.9 Growth curve of the isolated virus

The virus titres (TCID_50_) for each time point were assessed and calculated based on the Reed–Muench method to describe the viral growth kinetics. The virus isolates were briefly inoculated into the MDBK cells growing in the six-well plates at an MOI of 0.05. After incubation at 37°C for 1 h, the cells were washed twice with PBS. Two hundred microliters of MDBK cell supernatants were harvested at 24, 48, 72, 96, and 120 h and stored at −80°C. After measuring the mean titres of three independent measurements at each time and point, the growth curves were determined.

### 2.10 Whole-genome sequencing, sequence alignment, homology analysis, and phylogenetic tree construction

The sequencing of the extracted RNA samples from isolated strains were performed commercially from Shanghai TanPu Biotechnology Co., Ltd. The sequence differences between this isolate and all other BVDV strains obtained in GenBank were analyzed using DNA Star software 7.0 (26). Information on the reference BVDV strains was downloaded from GenBank. Phylogenetic analysis was performed based on genome-wide genes Mega software 6.0 was used to construct the phylogenetic tree (27).

### 2.11 Animal experiments

The animals had free access to food and water and were kept in a temperature-controlled room (24°C ± 0.5°C). This study has obtained approval from the Special Committee on Scientific Research and Academic Ethics and Morality of Inner Mongolia Agricultural University (Permit Number: [2020] 010) to ensure that the animal experimental procedures comply with ethical norms and biosafety requirements. Twenty 2–3 months old 1.5–2.2 kg New Zealand white rabbit females were divided into four groups: five CP-type BVDV-infected, five NCP-type BVDV-infected, five mixed-infection group, and five control group. After 7 days of rearing under normal conditions, and 1 ml of viral solution was injected into each of the CP-infected and NCP-infected groups, and 500 μl of each of the CP-type and NCP-type BVDV in the mixed-infection group via the marginal auricular vein (this time was marked as 1 day); saline was used as a substitute for the control group. The control group was replaced by saline. The New Zealand white rabbits were continuously infected for 3 days. Clinical symptoms were monitored daily.

### 2.12 Clinical symptoms, body temperature and body weight measurements

Monitoring the mental state, behavioral patterns, body weight, body temperature changes, and fecal consistency of the experimental rabbits under different groups following infection as important indicators of clinical alterations.

### 2.13 Replication kinetics of BVDV infected New Zealand white rabbits

According to the established experimental groups, lung, spleen, trachea, and small intestine samples were collected on day 18 post-infection to monitor the replication and spread of the virus in various tissues. Total RNA was isolated from the tissues using the AxyPrep™ Multisource Total RNA Miniprep Kit (Axygen, Corning, China). All surgical procedures adhered to the animal tissue protocol outlined in the manual. Total RNA extracted from 20 mg of tissue was reverse transcribed into cDNA using HiScript III Reverse Transcriptase (Vazyme, Nanjing, China) for nucleic acid detection.

### 2.14 Blood test

On days 0, 3, 6, 9, 12, 15, and 18 post-virus infection, we collected peripheral blood from New Zealand white rabbits using EDTA anticoagulant tubes via the marginal ear vein. Whole blood samples were analyzed using the fully automated blood cell analyzer (Procyte) from IDEXX Laboratories to perform a five-part complete blood count, which included parameters for white blood cells (WBC), lymphocytes (LYM), and platelets (PLT).

### 2.15 Histopathology of BVDV-infected New Zealand white rabbits

The lungs, spleen, trachea, and small intestine of New Zealand white rabbits after 18 days of infection were collected and placed in a 4% (v/v) paraformaldehyde fixative solution for histopathological analysis. The paraffin blocks embedded with fixed tissues or organs were serially cut into 5 mm sections and mounted on glass slides. The pathological sections stained with hematoxylin-eosin (HE) were then subjected to microscopic examination to evaluate the tissues' pathological damage.

### 2.16 Statistical analysis

Calculate and summarize statistical data to assess the overall data quality. All data were processed using IBM SPSS Statistics (version 22) for analysis, while graphs were created using GraphPad Prism software (version 8.0). *In vitro* experiments were performed independently at least three times, and the *t*-test was used to evaluate the statistical significance of the viral titers. Statistical significance was considered when the *p*-value was < 0.05, 0.01, or 0.001.

## 3 Results

### 3.1 Detection of BVDV in ear tissues samples

Overall, 103 samples were collected from 2021 to 2022, including 60 ear tissues from cattle without clinical symptoms and 43 anal swabs from cattle with clinical symptoms. Among the 60 ear tissues, 17 (28.3%) tested positive for BVDV nucleic acid. Three NCP strains of BVDV were isolated from them, with an isolation rate of 17.6%. Among the 43 anal swabs, 16 (37.2%) tested positive for BVDV nucleic acid. Three strains of BVDV were isolated from them, with an isolation rate of 18.75%, of which two were the NCP-type, with an isolation rate of 12.5%, and one was the CP-type, with an isolation rate of 6.25% (Table 1).

**Table 1:** Testing result of BVDV samples by RT-PCR.

	**Source**	**Sample**	**Number of samples**	**Number of positive nucleic acids**	**Positive rate (%)**	**Number of isolates**	**Separation rate (%)**
BVDV	Calves without clinical symptoms	Ear tissues	60	17	28.3	3	17.6
	Calves with clinical symptoms	Anal swab	43	16	37.2	3	18.75
Total			103	33	32.03	6	18.18

### 3.2 Isolation and identification of BVDV

**Figure 1:**
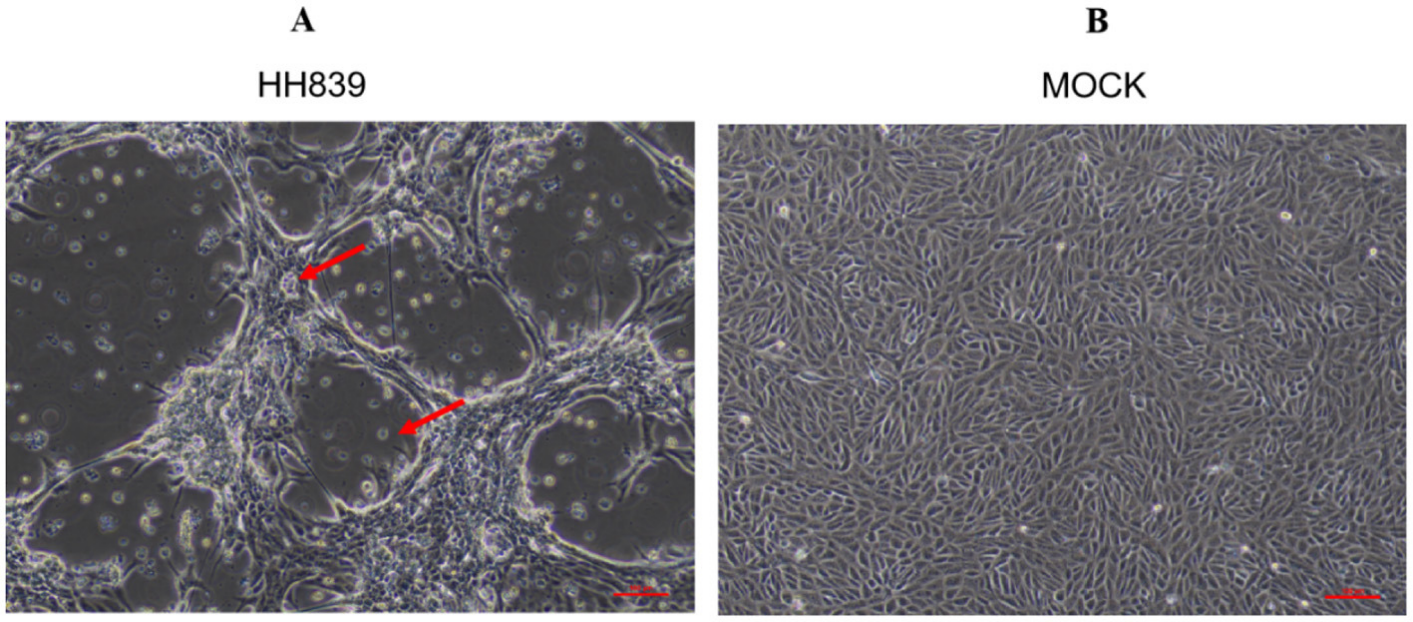
CPE caused by BVDV HH839 in MDBK cells at 96 h. **(A)** Shows MDBK cells infected with BVDV HH839, exhibiting clear cytopathic effects (CPE) such as rounding and detachment of cells. **(B)** Displays the mock control MDBK cells, which remain uninfected and show normal cell morphology without any signs of pathology.

The processed anal swab samples were inoculated into mono-layer MDBK cells; these cells were cultured in a 5% CO_2_ incubator at 37°C for 5 days and passed through three generations continuously in a blind manner. After one part of the virus solution was inoculated into MDBK cells, the cells were passed through the third generation in a blind manner. After 72 h of culture, the cells showed shrink-age, rounding, and wiredrawing (elongation or stretching of the cell membrane and cytoplasm), which are typical cytopathic effects associated with viral infection. After 96 h, a significant number of cells detached from the substrate and underwent cell death (Figure 1). Similar pathological effects were observed during each passage. Sequencing the whole genome of the cultured virus showed that it had high sequence homology with BVDV, and there was no match with other viruses. The whole BVDV genome was spliced, aligned, and analyzed to confirm that the virus was BVDV.

### 3.3 Electron microscopy observation of BVDV

**Figure 2:**
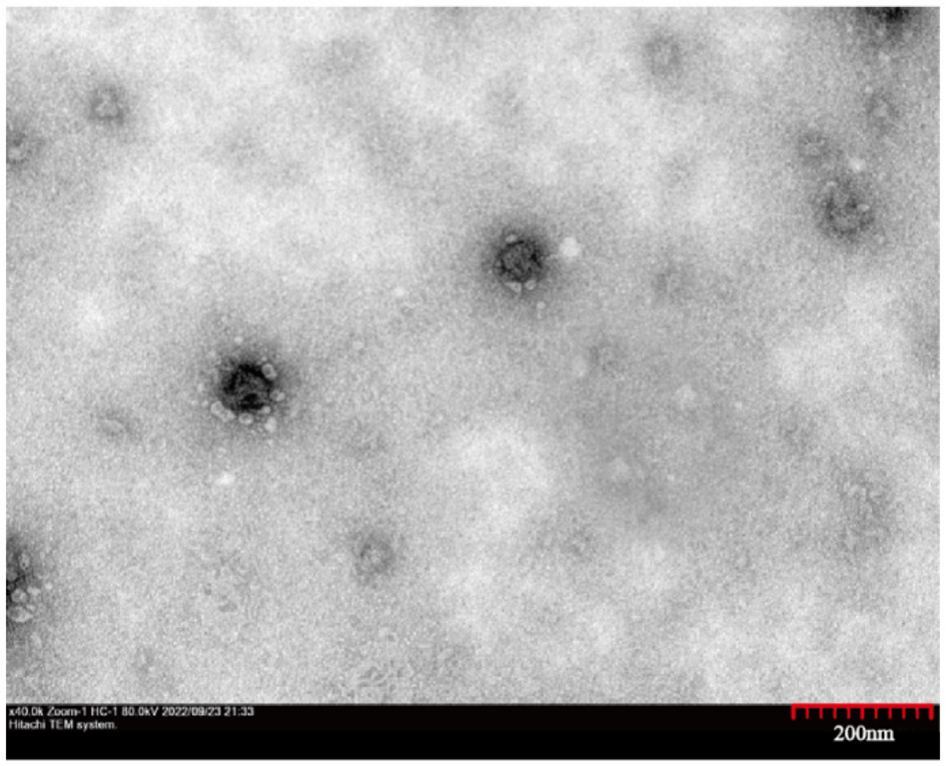
Transmission electron micrographs of the HH839. High magnification image showing the spherical morphology of HH839 virus particles, ~60 nm in diameter.

Virus morphology was analyzed using transmission electron microscopy. There were typical virus particles in MDBK cells with a diameter of ~60 nm (Figure 2), which conformed to the morphological characteristics of BVDV.

### 3.4 Direct immunofluorescence

**Figure 3:**
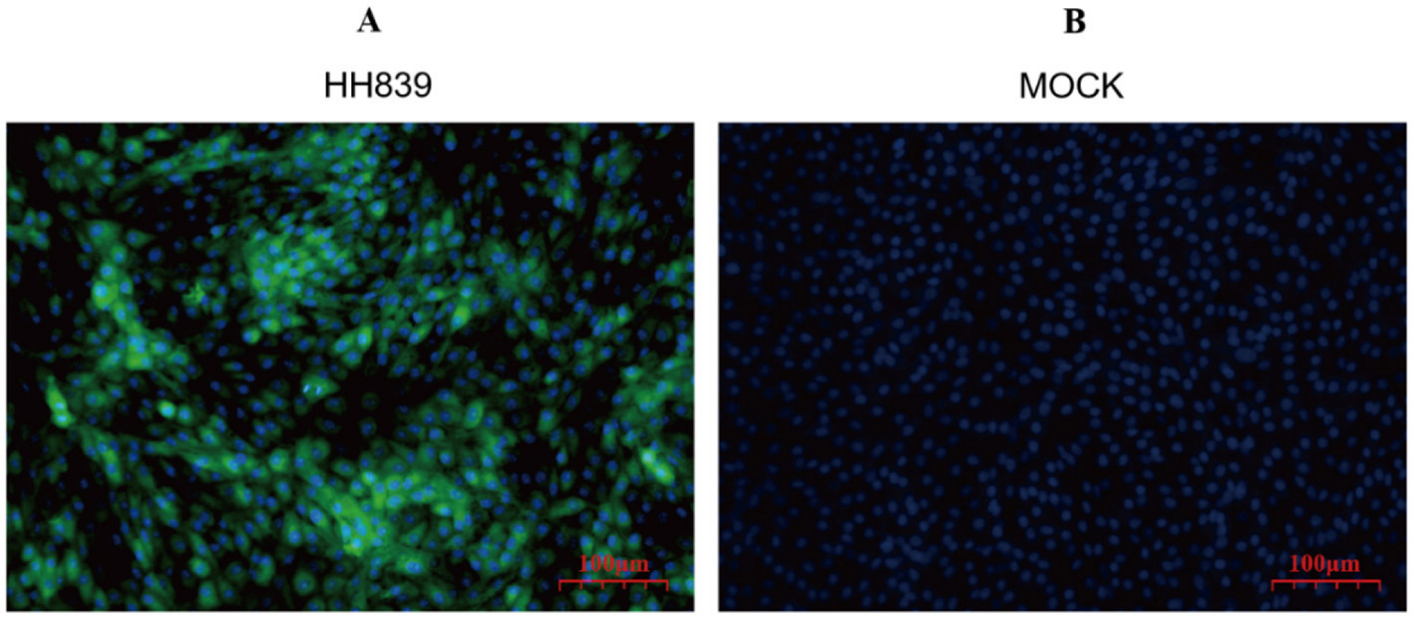
Immunofluorescence map of HH839. **(A)** Merged image of MDBK cells infected with the HH839 strain of BVDV, illustrating the presence of BVDV with green fluorescence localized in the cytoplasm, alongside blue fluorescence from DAPI staining of the nuclei, indicating cell integrity. **(B)** Control (mock) MDBK cells stained solely with DAPI, exhibiting blue fluorescence of the nuclei, confirming the absence of viral infection and highlighting the specificity of the immunofluorescence staining in **(A)**.

Direct immunofluorescence staining was performed 24 h after the HH839 strain of BVDV was infected with MDBK cells, as shown in Figure 3, BVDV was green after excitation and was located in the cytoplasm, and the nuclei of the cells after DAPI staining were blue.

### 3.5 Serum neutralization

Serum neutralization assay revealed the BVDV-positive standard serum could neutralized the isolated virus antigen, and cytopathy was not observed. After the isolated virus interacted with the BVDV-negative serum, the cells appeared cytopathic, further confirming the identity of the isolated virus as BVDV.

### 3.6 TCID_50_ titration of viral isolates

The TCID_50_ of the fifth generations strain of BVDV HH-839 was 10^5.23^/0.1 ml according to the Reed-Muench method (Table 2).

**Table 2:** Determination of the HH839 isolate TCID_50_.

**Virus dilution ratio**	**Positive wells**	**Negative wells**	**Cumulative of positive wells**	**Cumulative of negative wells**	**Proportion of number of CPE/%**
10^−1^	8	0	38	0	100
10^−2^	8	0	30	0	100
10^−3^	8	0	22	0	100
10^−4^	8	0	14	0	100
10^−5^	4	4	6	4	60
10^−6^	2	6	2	10	17
10^−7^	0	8	0	18	0
10^−8^	0	8	0	26	0

### 3.7 Growth curve of the isolated virus

**Figure 4:**
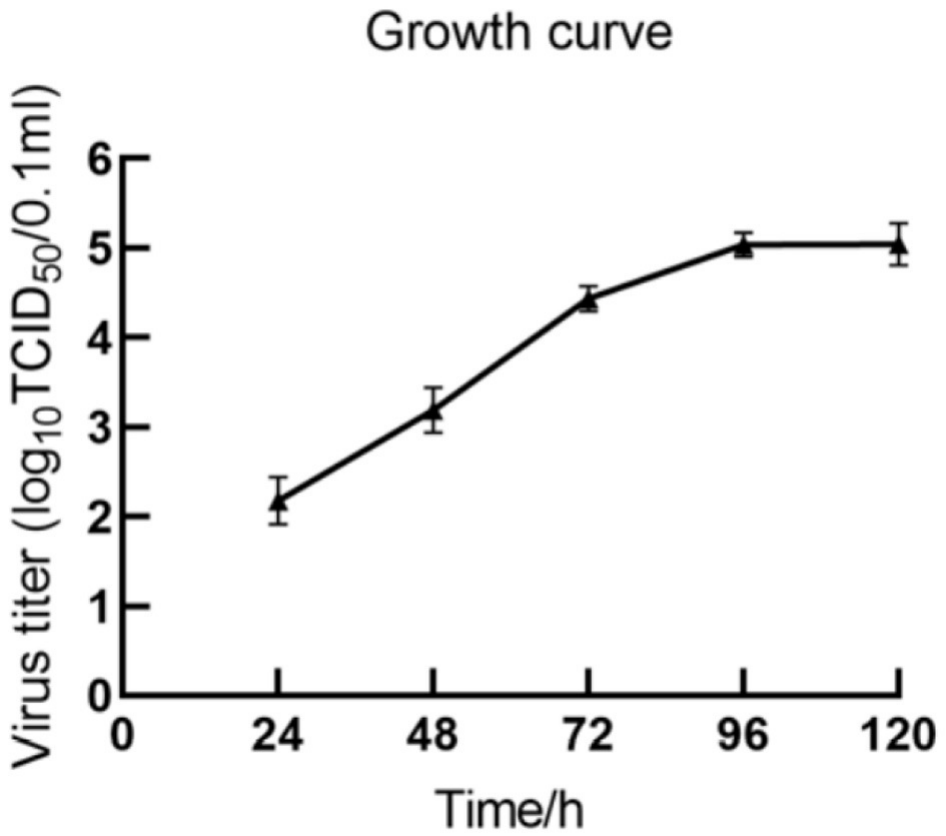
Growth curve of BVDV HH839 in MDBK cells.

The multi-step growth curve of the fifth-generation HH839 strain was further analyzed using MDBK cells. As shown in Figure 4, the viral load gradually increased starting from 24 h and reached a peak titer of 5.23 log_10_TCID_50_/0.1 ml at 96 h. Subsequently, it stabilized at 120 h. These results indicate that a BVDV strain was successfully isolated using MDBK cells.

### 3.8 Whole-genome sequencing, sequence alignment, and phylogenetic tree construction

**Figure 5:**
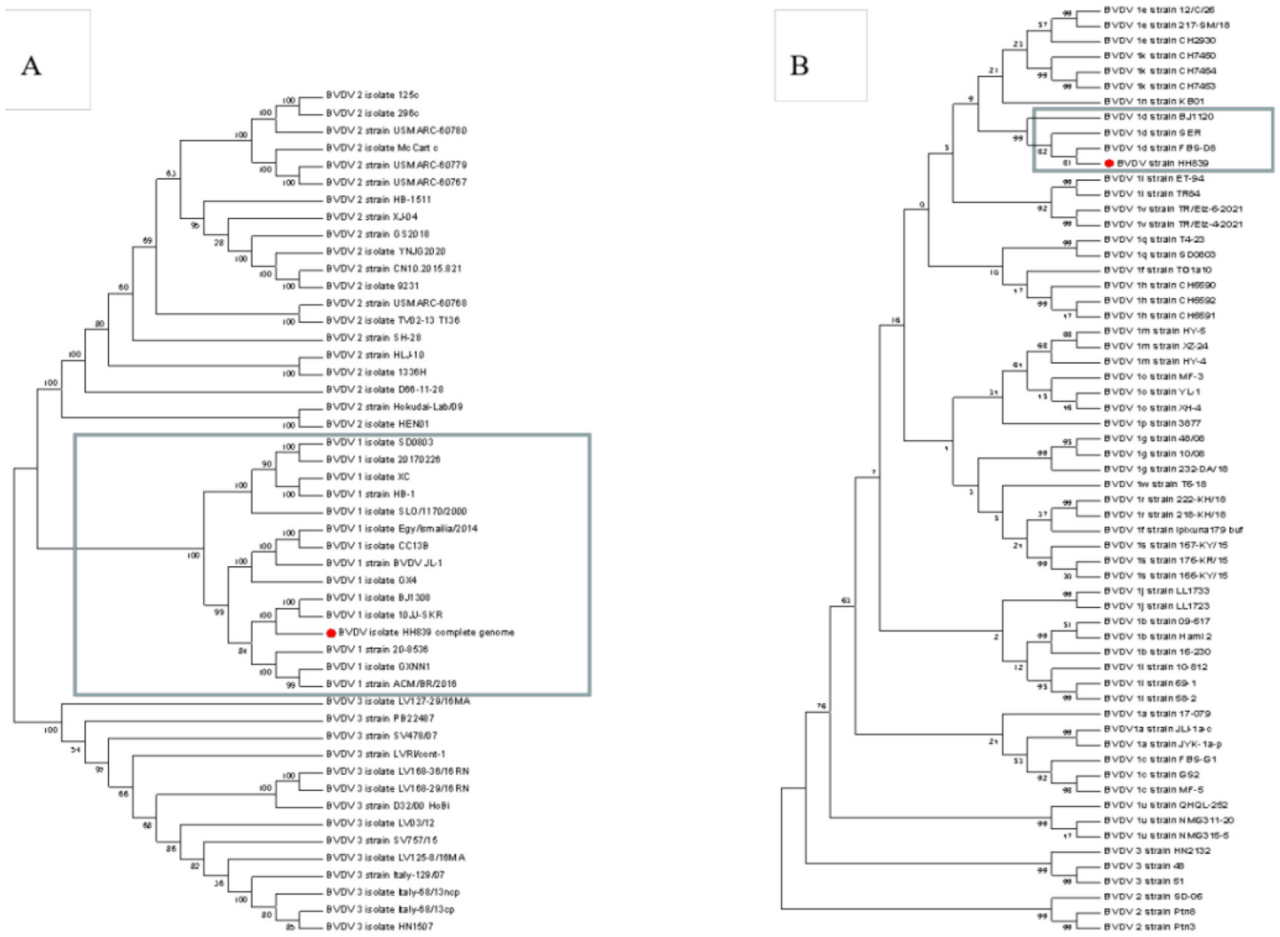
Phylogenetic analyses of the BVDV HH839 strain (GenBank accession number OP661170). Phylogenetic tree based on the full-length genome sequences of BVDV strains reported around the world. A neighbor-joining phylogenetic tree of 49 full-length BVDV genome classifying all the strains into three major clades (BVDV-1, BVDV-2, and BVDV-3), phylogenetic tree of 61 5′UTR BVDV genome classifying all the strains into different subtype (BVDV-1a, BVDV-1b—BVDV-1w). Phylogenetic trees were constructed using the neighbor—joining method with 1,000 bootstrap replicates in the MEGA 7.0 software. **(A)** The full-length of the BVDV HH839 strain, the GenBank accession numbers of the reference strains: JN400273.1, MK102095, MH166806, MZ188972, KX987157.1, KF772785.1, KR029825.1, KF501393.1, KJ689448.1, KC757383.1, KT951841.1, MT654137.1, MT079816.1, KX857724.1, KT832820.1, KT832821.1, KT832822.1, KT832823.1, KX096718.1, MG879027.1, MH806434.1, MH806435.1, MH806436.1, MH806437.1, MH806438.1, MW006485.1, MW528234.1, MW528235.1, MW168422.1, FJ527854.1, JF714967.1, HQ258810.1, MN527354.1, AB567658.1, MH410816.1, MH410815.1, MH410814.1, MH410813.1, MH410812.1, KY762287.2, KY767958.1, VKY683847.1, KU563155.1, KC297709.1, KJ627180.1, KJ627179.1, KC788748.1, and AB871953.1; **(B)** 5′UTR of the BVDV HH839 strain, the GenBank accession numbers of the reference strains: OM337791.1, MN248504.1, OM337788.1, MK684379.1, FJ621578.1, MN248500.1, JQ071527.1, MN513401.1, KY865373.1, MN513406.1, KF925510.1, MT179840.1, MW114079.1, MK381391.1, MH903552.1, MF977720.1, KX517783.1, JN715036.1, JN715004.1, MK381402.1, MH907214.1, MH907213.1, MH907212.1, MK248946.1, KF023369.1, KF023355.1, GU987138.1, GU987136.1, MH908076.1, MH908075.1, MH908072.1, MK347344.1, MH753470.1, KY865368.1, KJ578918.1, KY865367.1, GQ495676.1, KY865380.1, KY865379.1, KY865371.1, MW013504.1, MN417855.1, JN248727.1, MK381396.1, MK381392.1, MK168334.1, MK044825.1, MK044824.1, KJ578884.1, KJ578866.1, KJ578862.1, MZ686435.1, MZ686434.1, MN417892.1, EU034175.2, FJ795044.1, EU034173.1, MZ189735.1, MZ189734.1, and OK032116.1.

The whole-genome length of BVDV HH839 was 12,213 nt, including one 370-bp 5′UTR, a 11,693-nt multiprotein gene and a 150-nt 3′UTR. The CDS of HH839 was located between nucleotides 371 and 12,064. The 5′UTR sequences and whole-genome sequences of HH839 were compared with the representative BVDV reference strains in GenBank. BVDV HH839 shared 80.03%−98.30% sequence identity with the other strains at the whole genome level, sharing the highest identity (98.30%) with the BJ1308 strain. The phylogenetic tree was constructed from the sequences of HH839 5′UTR sequences and the reference strains in GenBank. The isolate was in the same evolutionary clade as the reference strains BJ1120, SER, and FBS-D8 in GenBank and were closely related and belong to the BVDV-1d subtype (Figure 5).

### 3.9 Clinical symptoms, body temperature and body weight measurements

**Figure 6:**
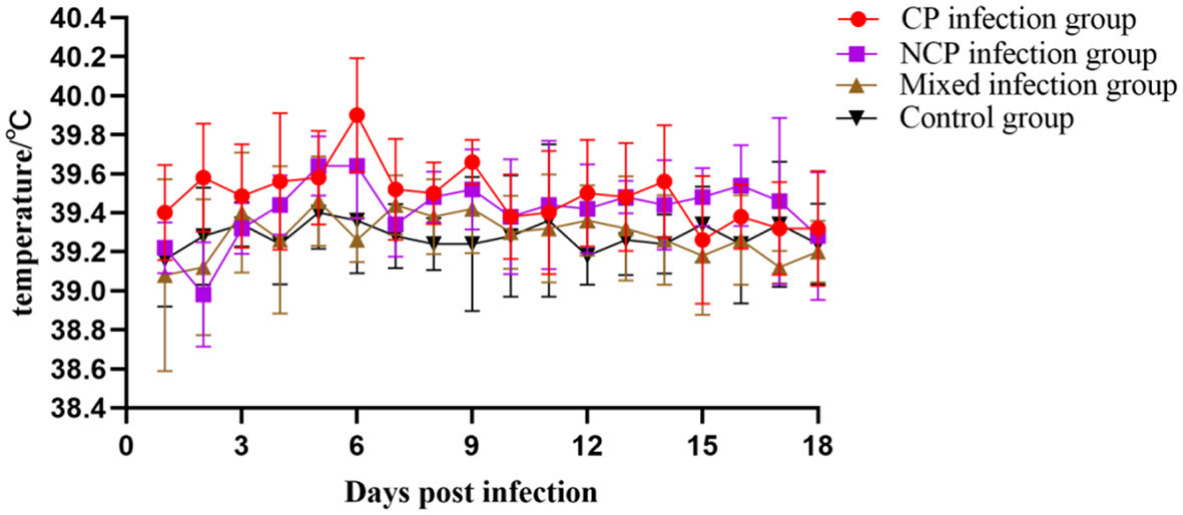
Body temperature changes of New Zealand White rabbits in each group.

**Figure 7:**
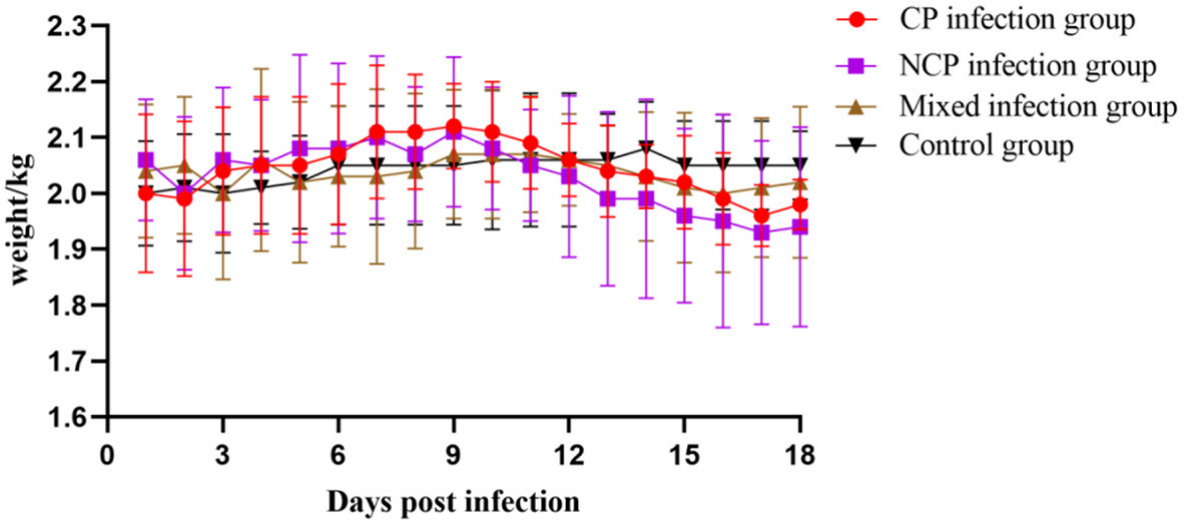
Weight changes of New Zealand White rabbits in each group.

The results showed that the activity of individual New Zealand white rabbits decreased and feeding was slightly reduced within 6 d after inoculation with bovine viral diarrhea virus (BVDV), and then gradually recovered to normal after 6 d. On the 10th day of infection, three rabbits in the NCP group showed symptoms of diarrhea, which decreased after 2 days. On the 13th day of infection, two rabbits in the CP group showed symptoms of diarrhea, which decreased after 2 days and one rabbit died on the 18th day of infection, while no symptoms of diarrhea were observed in the mixed group. After 10 days of infection, food and water intake decreased in all groups except the control group. The body temperatures of New Zealand white rabbits in each group were slightly elevated on the 5th day after inoculation, but fluctuated within the normal range, while the body temperatures of New Zealand white rabbits in the control group were normal (Figure 6). The body weight of New Zealand White rabbits in each infection group did not change significantly in the first 10 days after virus inoculation, and the body weight decreased slightly in the late stage of infection (Figure 7). The results showed that inoculation with bovine viral diarrhea virus had a slight pathogenic clinical manifestation in New Zealand white rabbits.

### 3.10 Replication kinetics of BVDV infected New Zealand white rabbits

Among the BVDV nucleic acid tests of all organs, the spleen positive rate was the highest, followed by the small intestine and lung, and the detection rate in the trachea was low (Table 3).

**Table 3:** Results of BVDV nucleic acid testing in different tissues after New Zealand white rabbits.

**Clusters**	**Samples**			
	**Lung**	**Spleen**	**Trachea**	**Intestine**
CP infection group	3/5	4/5	1/5	2/5
NCP infection group	1/5	3/5	0/5	3/5
Mixed infection group	2/5	5/5	2/5	2/5
Control group	0/5	0/5	0/5	0/5

### 3.11 Blood test results

Different biotypes of BVDV caused different changes in leukocyte, platelet, and lymphocyte counts. Leukocyte count changes tended to increase and then decrease in all infection groups, with the mixed-infection group starting to decline to a highly significant level compared to that in the control group on day 9 (*P* < 0.01). Platelet count in the CP infection group increased in the pre-infection period, reached a maximum value on day 12 to reach a significant level compared with that in the control group (*P* < 0.05) and then decreased. Platelet count in the NCP-type infection group and mixed-infection group decreased in the pre-infection period and began to rebound after reaching the lowest value on day 6 at a significant level compared with that in the control group (*P* < 0.05).

**Figure 8:**
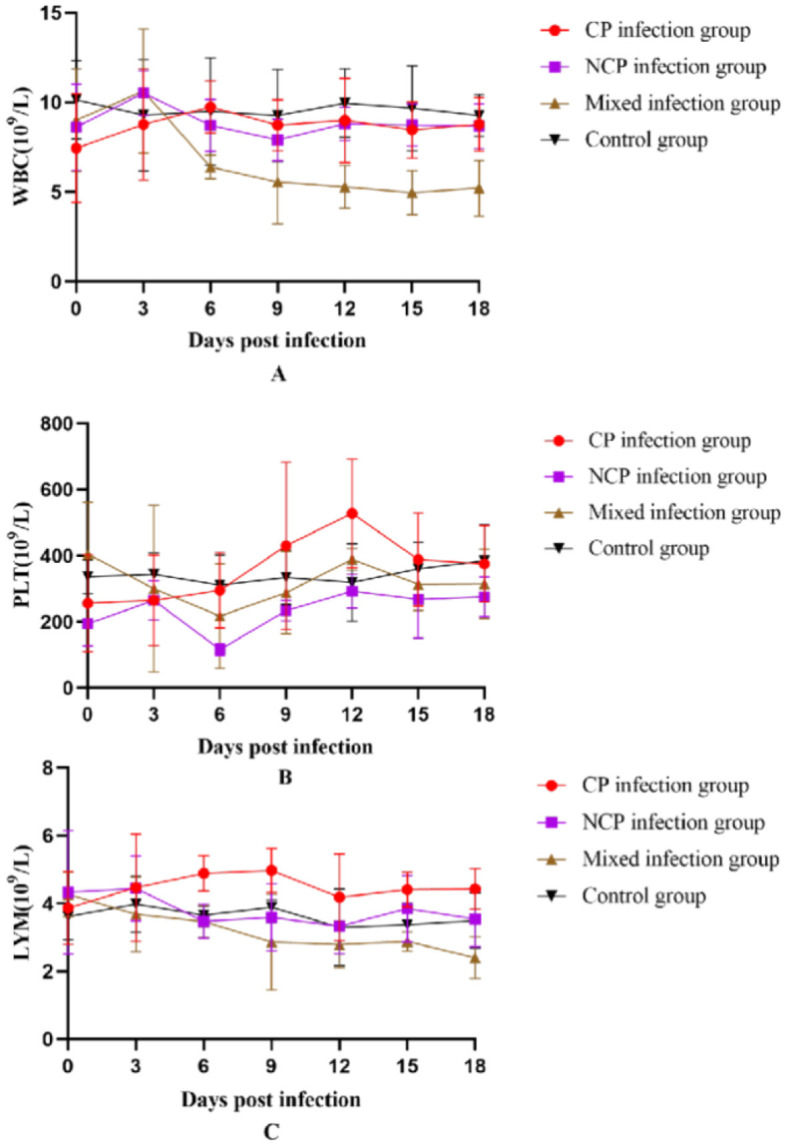
Number of white blood cells **(A)** White Blood Cell (WBC), **(B)** Platelet (PLT), **(C)** Lymphocytes (LYM) in the blood of New Zealand White rabbits in each group.

The CP-type infection group showed a slight increase in lymphocyte count that began to decline on day 6 after increasing to a significant level compared with that in the control group (*P* < 0.05); it showed a decreasing trend in the NCP-infection group since the infection. The changes in lymphocyte count in the mixed-infection group were in line with those in the NCP-infection group. However, the degree of decline was greater than that in the NCP-infection group; moreover, on day 18, it decreased to a significant level compared with that in the control group (*P* < 0.05). In conclusion (Figure 8), infection of New Zealand white rabbits with different bi-otypes of BVDV could cause different degrees of changes in leukocyte, platelet, and lymphocyte counts, but all of them fluctuated within the normal range.

### 3.12 Histopathology of BVDV-infected New Zealand white rabbits

**Figure 9:**
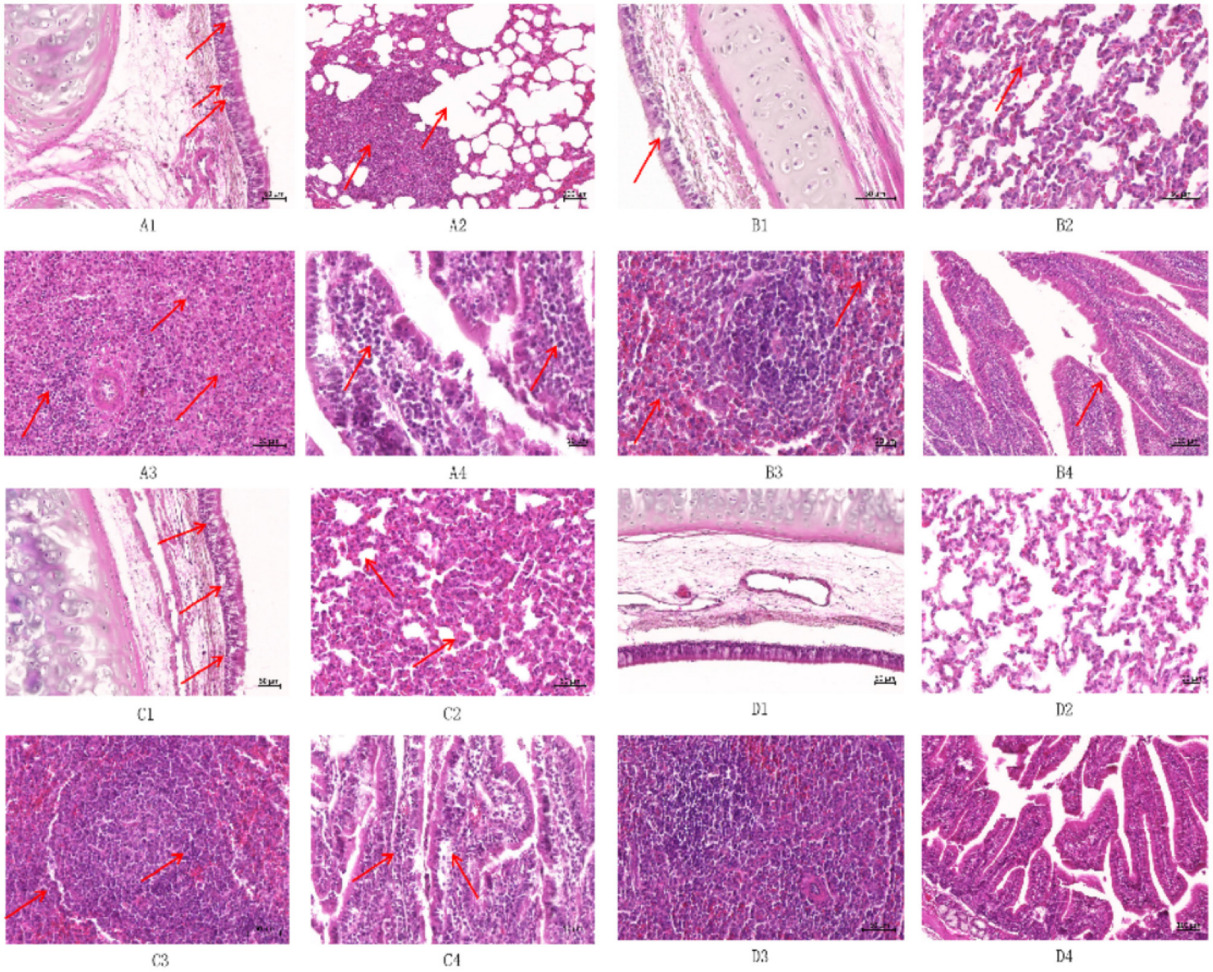
Histopathological observation of New Zealand White rabbits after infection (HE staining) ① **(A1)** Trachea of CP infection group (50×); **(A2)** Lung of CP infection group (50×); **(A3)** Spleen of the CP infected group (50×); **(A4)** Small intestine of CP infection group (50×); **(B1)** Trachea of NCP infection group (50×); **(B2)** Lung of NCP infection group (50×); **(B3)** Spleen of the NCP infected group (50×); **(B4)** Small intestine of NCP infection group (50×); **(C1)** Trachea of the mixed-infected group (50×); **(C2)** Lung of the mixed-infected group (50×); **(C3)** Spleens of the mixed-infected group (50×); **(C4)** Small intestine of the mixed-infected group (50×); **(D1)** Trachea of control group (50×); **(D2)** Lung of control group (50×); **(D3)** Spleen of control group (50×); **(D4)** Small intestine of control group (50×); ② The arrows indicate the lesion.

To explore the effects of different infection types on the body's tissues and organs, pathological sections were prepared by hematoxylin and eosin (HE) staining and observed under a light microscope to evaluate the degree of histopathological damage (Figure 9). The study involved a CP-type infection group, an NCP-type infection group, a mixed-infection group, and a control group. Multifaceted pathological observations were carried out on key parts such as the trachea, lungs, spleen, and small intestine. The results showed that the infection groups exhibited significantly different pathological characteristics from the control group in the above-mentioned parts, and the specific manifestations are as follows.

Tracheal epithelial cells in the CP-type infection group showed blister-like degeneration with scattered necrosis and nuclear condensation (Figure 9A1); tracheal epithelial cells in the NCP-type infection group had a small amount of blister-like degeneration without necrosis (Figure 9B1). Tracheal epithelial cells in the mixed-infection group showed typical blister-like degeneration, and there was an increase in lymphocyte and macrophage counts in the submucosal layer (Figure 9C1). The control group had an intact, structurally sound tracheal mucous layer, with intact and undamaged epithelium (Figure 9D1).

Lymphocyte proliferation and infiltration were present in the interstitium of the lungs in the CP-type infection group, showing typical mild-to-moderate interstitial pneumonia with type II epithelial cell hyperplasia; compensatory emphysema was clearly present at the borders of the inflammatory foci (Figure 9A2). Lymphocyte count increased in the lungs in the NCP-type infection group compared with those in the control group, and there were no obvious pathologic changes (Figure 9B2); alveolar septa of the lungs in the mixed-infection group widened, showing moderate interstitial pneumonia, with an increase in macrophage, lymphocyte, and erythrocyte counts, as well as type II epithelial cell hyperplasia (Figure 9C2); there was no infil-tration of inflammatory cells in bronchial tubes and alveoli of the control group, which showed a normal pattern (Figure 9D2).

The white and red medullas of the spleen in the CP-type infection group were not clearly defined, with a marked decrease in the volume of the white medulla and an increase in the volume of the red medulla, which was filled with many erythrocytes (Figure 9A3). The white and red tunnel medullas of the spleen in the NCP-type infection group were well defined, with an occasional de-crease in lymphocyte counts in the white medulla but no necrosis, as well as an increase in erythrocyte counts at the red medulla (Figure 9B3). The splenic lymphocytes of the germinal centers in the mixed-infection group were markedly reduced, and there was a scattering concentration of the nuclei of lymphocytes, with occasional necrosis, and an increase in the number of reticulocytes (Figure 9C3). The white and red medullas of the control group had intact structures, with no injury (Figure 9D3).

The epithelial cells of the small intestinal mucosa in the CP-type infection group were exfoliated but not necrotic, and the structure was relatively intact, with typical lymphocytic infiltration in the lamina propria (Figure 9A4). The epithelial cells of the small intestinal epithelium in the NCP-type infection group were slightly exfoliated but structurally intact, with no obvious pathologic damage (Figure 9B4). The lymphocyte, macrophage, and plasma cell count in the lamina propria of the small intestinal mucosa increased in the mixed-infection group (Figure 9C4), and the intestinal wall was structurally intact in the control group, with the villous epithelial cells intact (Figure 9D4).

## 4 Discussion

The isolation and identification of CP-type BVDV in Inner Mongolia have been rarely documented. This study successfully isolated and identified BVDV from calves based on clinical signs. BVDV can cause various clinical syndromes, including respiratory issues, reproductive dysfunction, immunosuppression, persistent infection, and mucosal diseases (28). It mainly infects cloven-hoofed mammals, primarily targeting cattle, while other species such as camels, deer, sheep, goats, and pigs may show seroconversion (29–31). The virus has been endemic in Chinese cattle, resulting in significant economic losses for the livestock industry. BVDV is classified into biotypes and species, with biotypes determined by the presence or absence of cytopathic effects (CPE) in infected cell cultures, distinguishing cytopathic (CP) and noncytopathic (NCP) subtypes (32). The following subtypes are now prevalent in China: 1a-1d, 1m-1q, and 1u (14). Most BVDV strains isolated in China from 2004 to 2020 belong to the BVDV-1 species; three subtypes (BVDV-1a, c and m) accounted for 80.67% of BVDV-1 reported in China (15). The HH839 strain from this study demonstrated the highest nucleotide homology with the BJ1308 strain and classified as BVDV-1d subtype. This represents the first report of this subtype in the Hohhot region, raising concerns of an epidemic possibly linked to allopatric introduction.

The high mutation rate of bovine viral diarrhea virus (BVDV), an RNA virus, contributes to genetic variability during replication and evolution (33). These mutations can significantly influence viral adaptability and pathogenicity, underscoring the importance of continued BVDV isolation, identification, and genetic characterization. Such efforts enable timely formulation of targeted prevention and control strategies based on epidemiological trends and genetic variations. Currently, no specific antiviral drugs are available for BVDV, making vaccination the primary preventive measure. While inactivated vaccines are predominantly used, their efficacy remains suboptimal. Consequently, the identification of BVDV strains with strong immunogenic properties holds great significance for advancing vaccine development.

While BVDV is typically thought to have a low likelihood of infecting non-ungulate species, several studies suggest that rabbits can be susceptible to this virus. However, doubts remain about the validity of using rabbits as experimental models. Therefore, it is essential to investigate the infectious mechanisms of New Zealand White rabbits as potential BVDV hosts. Bu et al. infected rabbits via intraperitoneal injection, confirming successful artificial infection using PCR (34), despite the absence of significant clinical alterations after infection with Henan isolates. Yang demonstrated that the BVDV-1c subtype could infect domestic rabbits through artificial inoculation (35), leading to substantial pathological damage and the generation of BVDV-specific antibodies. Subsequently, Xiang Siyi and his team employed intranasal, oral, and intravenous infection methods on lactating rabbits. Autopsy and PCR detection on the intestinal mucosa seven days post-infection revealed BVDV (36). This denotes that the virus resulted in pathogenic effects on lactating rabbits after the initial infection. The variations in results might be attributed to differences in infection methods, virus dosages, and rabbit breeds. Research from 2014 confirmed that New Zealand White rabbits could be infected through intravenous methods or natural exposure to contaminated hay, with BVDV detected in multiple organs and notable lymphatic tissue changes (37). To conclusively evaluate the potential of rabbits as experimental BVDV hosts, this study involves the artificial infection of New Zealand White rabbits.

This study successfully demonstrated the feasibility of using New Zealand White Rabbits as experimental models for BVDV infection. Through systematic evaluation of clinical symptoms, virological detection, hematological analysis, and histopathological examination, we confirmed that intravenous inoculation of BVDV could induce characteristic pathological changes consistent with natural infection. Key findings include the detection of BVDV RNA in multiple tissues, with the spleen showing the highest positivity rate, suggesting its role as a primary target organ. The observed fluctuations in body temperature and weight loss, coupled with significant hematological changes such as leukopenia and thrombocytopenia, further corroborated the successful establishment of infection. Histopathological examination revealed varying degrees of tissue damage in critical organs, highlighting the systemic impact of BVDV infection.

Research indicates that discrepancies exist in the isolation rate and clinical symptoms of different BVDV biotypes. The NCP subtype isolates significantly outnumber the CP, which is consistent with findings that suggest NCP biotypes are often associated with subclinical infections, while CP biotypes are linked to more severe clinical manifestations. This highlights the importance of understanding the relationship between these biotypes and the spectrum of diseases they can cause, as well as their implications for diagnosis and treatment (38). Cattle infected with the NCP biotype are typically transiently infected, while persistent infection (PI) cases are more frequently isolated through control programs. Notably, PI occurs via *in utero* transmission from dams, whereas transient infections are acquired through respiratory exposure. Conversely, cattle with necropsy lesions of enteritis/colitis with systemic lesions had more CP strains than NCP strains, which is attributed to the progression to mucosal disease in persistently infected animals (39). Certain scholarly references have noted that super-infections can instigate an outbreak of mucosal disease, always leading to fatality. Notably, our results underscored the distinct pathogenic characteristics of different BVDV biotypes. The predominance of NCP subtype isolates aligns with previous reports indicating their association with subclinical infections, while the CP subtype was linked to more severe clinical manifestations. The exacerbated hematological and histopathological changes observed in super-infected rabbits suggest a synergistic effect that warrants further investigation.

Despite these contributions, several limitations must be acknowledged. The short observation period restricts the comprehensive assessment of long-term sequelae. Future studies should aim to optimize the infection models to more accurately mimic natural transmission routes and explore the underlying mechanisms of BVDV-induced immunopathology. Such efforts will enhance our understanding of BVDV pathogenesis and inform the development of effective diagnostic and therapeutic strategies.

## 5 Conclusions

This study isolated the HH839 strain from calves with severe diarrhea and respiratory diseases in Hohhot, Inner Mongolia. Through clinical and laboratory analysis, it was identified as a cytopathic BVDV-1d strain, offering key references for BVDV research. Using New Zealand white rabbits, we investigated BVDV pathogenicity. The super-infected group (CP and NCP types) showed more obvious pathogenic signs than single—infected groups. Our findings deepen the understanding of BVDV's biology and pathogenicity. They also provide a theoretical basis for vaccine development, diagnostic improvement, and control strategy formulation, with potential benefits for the livestock industry.

## Data Availability

The original contributions presented in the study are publicly available. This data can be found here: https://www.ncbi.nlm.nih.gov/nuccore/OP661170.1//accessionnumber:OP661170.
